# Transgenic mice with ectopic expression of constitutively active TLR4 in adipose tissues do not show impaired insulin sensitivity

**DOI:** 10.1002/iid3.162

**Published:** 2017-08-04

**Authors:** Kikumi D. Ono‐Moore, Ling Zhao, Shurong Huang, Jeonga Kim, Jennifer M. Rutkowsky, Ryan G. Snodgrass, Dina A. Schneider, Michael J. Quon, James L. Graham, Peter J. Havel, Daniel H. Hwang

**Affiliations:** ^1^ Department of Nutrition University of California Davis California; ^2^ Western Human Nutrition Research Center Agricultural Research Service, USDA‐ARS Davis California; ^3^ Department of Nutrition University of Tennessee Knoxville Tennessee; ^4^ Division of Endocrinology, Diabetes, and Metabolism Department of Medicine, University of Alabama Birmingham Alabama; ^5^ Department of Cardiovascular Medicine University of California Davis California; ^6^ Division of Endocrinology, Diabetes and Nutrition University of Maryland, School of Medicine Baltimore Maryland

**Keywords:** TLR4, transgenic, adipose tissue, insulin sensitivity

## Abstract

**Introduction:**

Chronic low‐grade inflammation is associated with obesity and diabetes. However, what causes and mediates chronic inflammation in metabolic disorders is not well understood. Toll‐like receptor 4 (TLR4) mediates both infection‐induced and sterile inflammation by recognizing pathogen‐associated molecular patterns and endogenous molecules, respectively. Saturated fatty acids can activate TLR4, and TLR4‐deficient mice were protected from high fat diet (HFD)‐induced obesity and insulin resistance, suggesting that TLR4‐mediated inflammation may cause metabolic dysfunction, such as obesity and insulin resistance.

**Methods:**

We generated two transgenic (TG) mouse lines expressing a constitutively active TLR4 in adipose tissue and determined whether these TG mice would show increased insulin resistance.

**Results:**

TG mice fed a high fat or a normal chow diet did not exhibit increased insulin resistance compared to their wild‐type controls despite increased localized inflammation in white adipose tissue. Furthermore, females of one TG line fed a normal chow diet had improved insulin sensitivity with reduction in both adiposity and body weight when compared with wild‐type littermates. There were significant differences between female and male mice in metabolic biomarkers and mRNA expression in proinflammatory genes and negative regulators of TLR4 signaling, regardless of genotype and diet.

**Conclusions:**

Together, these results suggest that constitutively active TLR4‐induced inflammation in white adipose tissue is not sufficient to induce systemic insulin resistance, and that high fat diet‐induced insulin resistance may require other signals in addition to TLR4‐mediated inflammation.

## Introduction

Toll‐like receptors (TLRs) can induce innate immune responses by recognizing invariant pathogen‐associated molecular patterns, leading to activation of downstream signaling pathways and the expression of diverse arrays of proinflammatory marker gene products that are required for host defense against invading pathogens. In addition, TLRs can be activated by endogenous molecules derived from tissue injury or stress and saturated fatty acids (SFAs) to elicit sterile inflammation 1, 2, 3, 4, 5. Results from genetic, clinical, and biochemical studies suggest that TLR‐mediated inflammation is an important determinant in modifying the risk of the development of many chronic diseases, including diabetes and cardiovascular disease 6.

Our previous studies revealed that SFAs can activate TLR4‐, TLR2‐, and NODs‐mediated signaling pathways in vitro 1, 2, 4, 5, 7. Activation of TLRs or NODs by SFAs can induce activation of downstream signaling molecules, including IKKβ and JNK, leading to impairment of insulin signaling pathways. Such effects mechanistically link TLR‐induced inflammation to insulin resistance (IR) in adipocytes and muscle cells 8, 9, 10, 11, 12, 13, 14. In addition, TLR pathway induced cytokines, such as TNF‐α and IL‐6, are also known to impair insulin signaling 15, 16. TLR4‐mediated ceramide production can suppress glucose uptake and interfere with the insulin signaling pathway 17. Furthermore, the effects of SFA‐induced TLR4 activation on IR has been studied using TLR4 knockout (KO) mice or TLR4 loss‐of‐function mutant mice. Knock‐out or loss‐of‐function of TLR4 or TLR2 and TLR4 double KO mice are protected from high fat diet (HFD)‐induced inflammation and IR in white adipose tissue (WAT) 18, 19, 20. TLR2 KO mice also showed reduced WAT inflammation, and greater insulin‐stimulated glucose uptake in WAT stromal vascular cells compared to the wild‐type (WT) controls 21. In addition, administration of the TLR4 ligand LPS induces inflammation and IR in mice and humans 22, 23, 24, 25. TLR4 is ubiquitously expressed and thus, endogenously expressed TLR4 cannot be activated in an organ‐specific manner by LPS. Since saturated fatty acids or LPS can activate multiple signaling pathways, specific activation of TLR4 in a ligand independent manner would be a more appropriate experimental model to determine whether TLR4‐mediated inflammation is sufficient to induce metabolic dysfunction. Therefore, we generated transgenic mice ectopically expressing a constitutively active TLR4 driven by aP2 promoter and evaluated metabolic changes in the transgenic mice compared to their littermate controls under a HFD and a normal chow diet (NCD).

We found that constitutive activation of TLR4 signaling pathway in adipose tissues was not sufficient to impair insulin sensitivity, regardless of diet. Rather, females of one line of TG mice fed a NCD exhibited improved insulin sensitivity and decreased adiposity. These results suggest that enhanced TLR4‐mediated inflammation in adipose tissue alone does not lead to impairment of systemic insulin sensitivity in mice, and that TLR4‐mediated inflammation may be necessary but not sufficient to induce the development of IR.

## Results

### ΔTLR4 transgene under aP2 promoter is specifically expressed in adipose tissue and bone marrow derived macrophages (BMDM)

**Figure 1:**
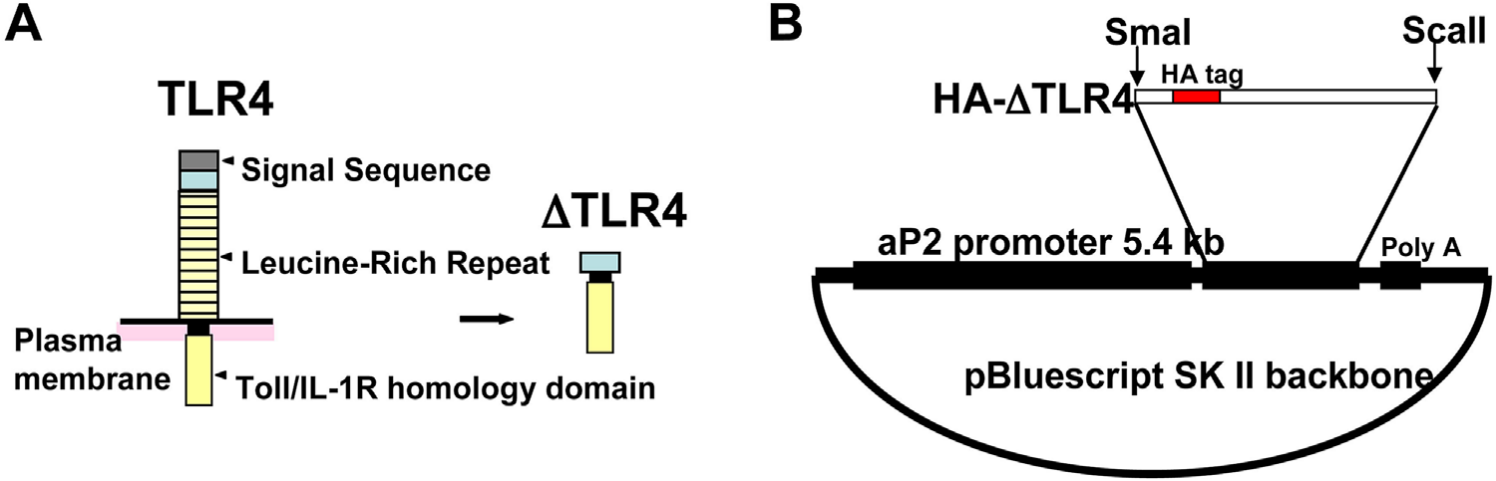
Generation of the HA‐ΔTLR4 construct. Constitutively active TLR4 (ΔTLR4) was generated by deleting leucine‐rich repeats in the N‐terminus from the full length TLR4 (A). ΔTLR4 was tagged with the hemagglutinin (HA) tag and was ligated downstream of aP2 promoter followed by a polyadenylation signal sequence of bovine growth hormone to generate aP2‐ΔTLR4, as shown in (B).

**Figure 2:**
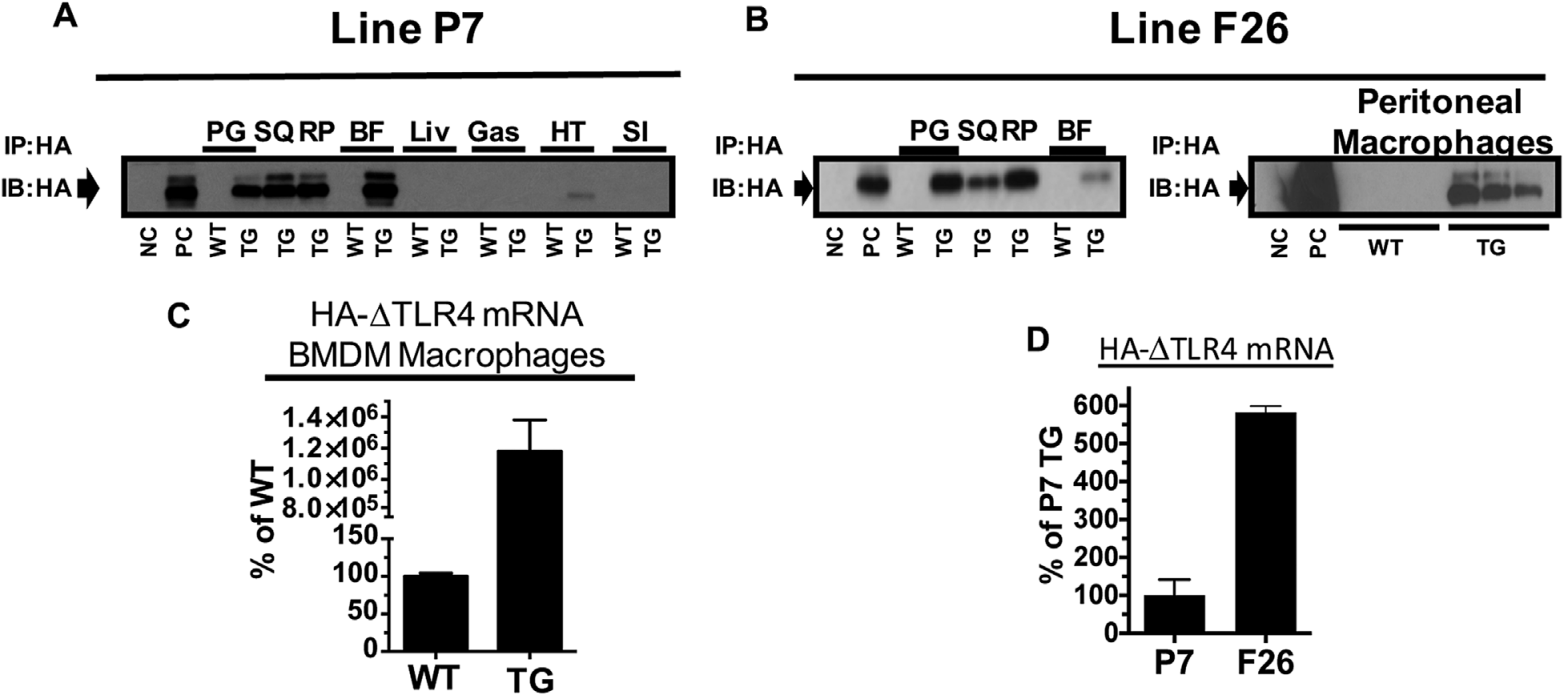
Expression of HA‐ΔTLR4 in fat pads and macrophages in the transgenic mice. (A and B) Protein expression of HA‐ΔTLR4 in various fat pads, tissues, or peritoneal macrophages from two lines were analyzed by immunoprecipitation. (C) mRNA expression of HA‐ΔTLR4 in the BMDM of line P7. (D) Line F26 has higher HA‐ΔTLR4 mRNA expression than that of the P7 line in perigonadal (PG) fat. Data = Mean ± SEM (*n* = 5). The experiments have been performed more than three times. The representative results are shown. Two tailed Student's *t*‐tests were conducted (WT vs. TG). *p < 0.05; **p < 0.01; ***p < 0.001. BF, brown fat; BMDM, bone marrow derived macrophages; Gas, gastrocnemius; HT, heart; Liv, liver; NC, negative control HEK293T cell lysate; PC, positive control (cell lysate of HEK293T cells transfected with HA‐ΔTLR4 expression plasmid); RP, retroperitoneal fat; SI, small intestine; SQ, subcutaneous fat; TG, transgenic mice; WT, wild‐type mice.

Two TG founder mice that express ΔTLR4 transgene were generated (P7 and F26 line). Schematic designs of the transgene under aP2 promoter (aP2‐ΔTLR4) were depicted in Figure 1. HA‐tagged ΔTLR4 protein was detected in various WAT depots (PG, RP, and SQ), BAT, and peritoneal macrophages (Fig. 2A and B). As expected, ΔTLR4 protein was not detected in the liver, gastrocnemius, small intestine, or brain and only small amount detected in cardiac muscle. Moreover, transgene mRNA was detected in BMDM of the P7 line (Fig. 2C). The transgene expression profile is consistent with reported endogenous aP2 expression in adipocytes 26, macrophages 26, and cardiomyocytes 27. When the transgene expression levels are compared, line F26 had an expression level that is around fivefold higher than that of line P7 (Fig. 2D). Therefore, we have chosen to focus on the P7 line, which may mimic the low‐grade inflammation seen in obesity. All data presented are from the P7 line unless otherwise indicated. Data from line F26 are provided in the Supplemental Figures.

### Constitutive activation of TLR4 under aP2 promoter decreases food intake, total body and fat pad weights, and muscle weight in female mice fed a NCD, but not a HFD

**Figure 3:**
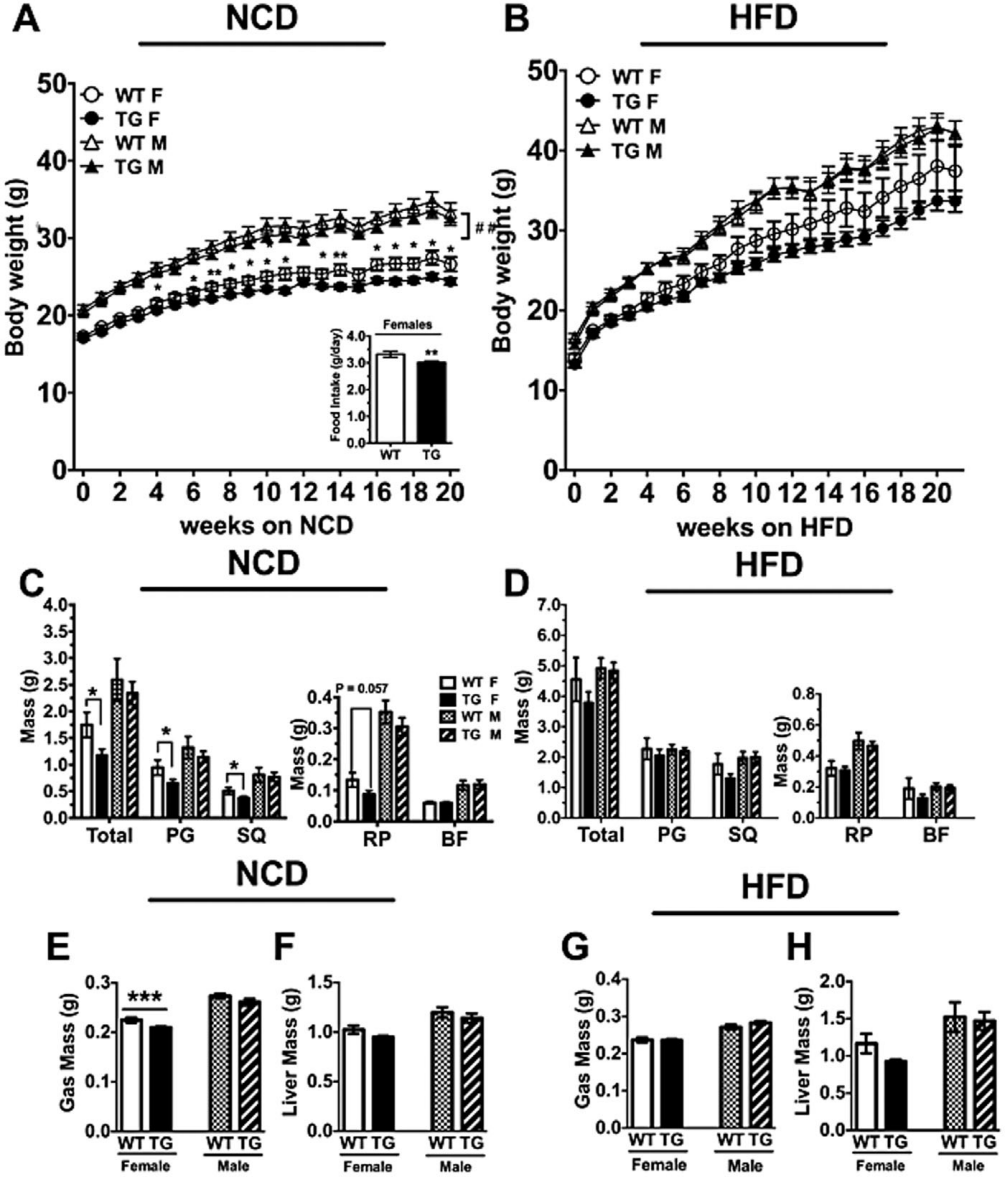
Body weight over time and terminal fat pad weights in TG mice compared with the WT littermate controls. (A and B) male (M) and female (F) TG and their WT littermate controls were fed either a NCD or HFD from ∼5 weeks of age and body weights recorded over 20 weeks on diet. The data were analyzed by ANOVA with repeated measures of genotype × time. ^##^ *p* < 0.01. (C and D) Tissue weights of various fat pads were measured at termination. RP and BF data are on separate graphs to better display the differences. (E–H) Gastrocnemius (gas) and liver weights from mice on the NCD (E and F) or HFD (G and H) were weighed. BF, brown fat; Gas, gastrocnemius; PG, perigonadal fat; RP, retroperitoneal fat; SQ, subcutaneous fat; TA, sum of total adipose. Data = Mean ± SEM (*n* = 7–27). A few error bars are hidden by the symbols. Two tailed Student's *t*‐tests were conducted (WT vs. TG). *p < 0.05; **p < 0.01; ***p < 0.001.

To study the effects of constitutively active TLR4 in adipose tissue on the development of obesity, inflammation and insulin resistance, transgenic mice, and their wild‐type littermate controls were put on either a normal chow diet (NCD) or a high fat diet (HFD). On the NCD diet, P7 female TG mice had a decrease in body weight starting at 4 weeks compared to their WT littermates (Fig. 3A). At termination (25 weeks of age), P7 female TG mice had gained 21% less weight than their WT littermates (time × genotype, *p* < 0.01 with ANOVA with repeated measures). When fed the HFD, P7 female mice also had less body weight, but the difference did not reach statistical significance (time × genotype, *p* = 0.127 with ANOVA with repeated measures, Fig. 3B).

P7 male mice fed the NCD or the HFD did not show significant differences in body weights (time × genotype, *p* = 0.941 and *p* = 0.987, respectively, ANOVA with repeated measures) (Fig. 3A and B). Furthermore, P7 female TG mice fed the NCD consumed less food than their WT littermates (WT: 3.32 ± 0.117, TG: 3.01 ± 0.054 g/day) (*p* < 0.01), as measured from 17th week on the diet to the end of the study. However, the food intake of P7 male mice was not different from that of the WT littermates during this period (WT: 3.48 ± 0.10, TG: 3.45 ± 0.11 g/day). P7 female and male TG mice fed the HFD showed no significant difference in food intake either (data not shown).

In agreement with body weight data, total adipose tissue weight (sum of collected WAT and BAT depots) of P7 female TG mice fed the NCD was smaller compared to their WT littermates. P7 female TG perigondal (PG) and subcutaneous (SQ) fat pads weighed significantly less than their WT littermates. P7 female TG retroperitoneal (RP) fat pads tended to weigh less than their WT littermates, but did not reach statistical significance (*p* = 0.057, Fig. 3C). In addition to decreases in adipose tissue masses, the gastrocnemius of P7 female TG mice weighed significantly less than their WT littermates (WT: 0.2246 ± 0.005 g, TG: 0.2096 ± 0.002 g; *p* < 0.05) (Fig. 3E). Neither P7 male TG mice fed the NCD or the HFD showed significant differences in the masses of either adipose depots (Fig. 3C and D) or gastrocnemius (Fig. 3E and G) when compared to their same sex littermates. Additionally, P7 female and male WT and TG livers weighed the same compared to their same sex littermates, regardless of the diet (Fig. 3F and H).

Body weights and fat tissue weights on either NCD or HFD were also measured in the second line of transgenic mice, line F26. There were no differences in body weight gain between the TG and WT littermates in both sexes when the mice were fed a HFD (Supplemental Fig. S1B) or between the female TG and WT mice when fed a NCD (Supplemental Fig. S1A). At termination, there were no differences in the fat pad weights in the female mice fed the NCD (Supplemental Fig. S1C) or males and females fed the HFD (Supplemental Fig. S1D).

### Constitutive activation of TLR4 under aP2 promoter does not impair systemic insulin sensitivity

**Figure 4:**
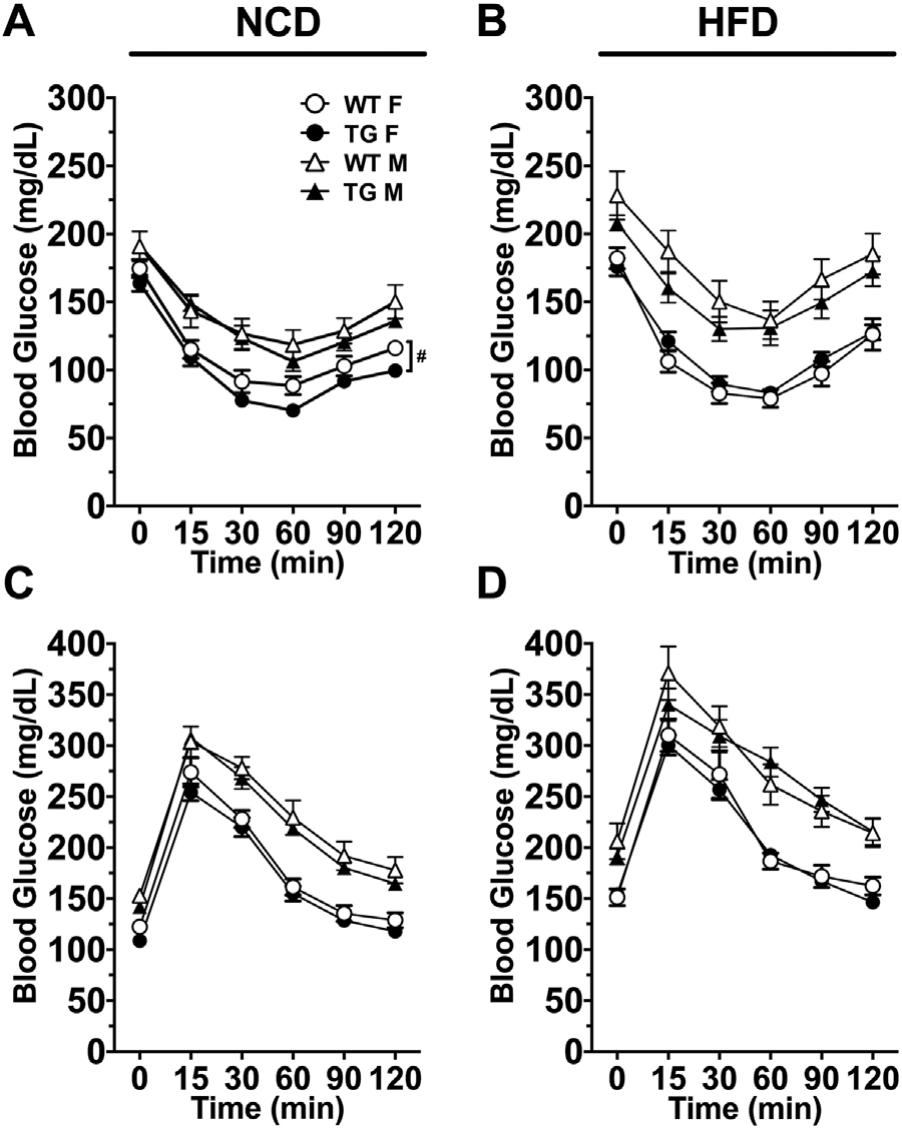
Female P7 TG mice fed the NCD were more insulin sensitive than their WT littermate controls. (A and B) Insulin tolerance tests were completed on TG mice compared to their WT controls in NCD and HFD, respectively. (C and D) Glucose tolerance tests from TG mice and WT controls fed NCD and HFD, respectively. Data = Mean ± SEM (*n* = 7–27). (A–D) ANOVA with repeated measures were used. ^#^ *p* < 0.05.

We examined the effects of transgene expression on insulin and glucose sensitivities. P7 female TG mice fed the NCD were more insulin sensitive than their WT littermates as measured by insulin tolerance tests (ITT) (genotype *p* < 0.05, ANOVA with repeated measures) (Fig. 4A). P7 male TG mice fed the NCD showed no significant differences in insulin sensitivity compared with their WT littermates (Fig. 4A). Under the NCD, male mice showed poor insulin sensitivity compared to their female counterparts fed the NCD (2‐way ANOVA, *p* < 0.05, sex, *p* < 0.001, no interaction) (Fig. 4A). Under the HFD, there were no differences in insulin sensitivities between the TG and WT mice of both sexes and male mice showed poor insulin sensitivity compared to their female counterparts (2‐way ANOVA; time, *p* < 0.001; sex, *p* < 0.001; no interaction) (Fig. 4B). On the other hand, WT and TG mice of both sexes showed no significant differences in glucose tolerance as measured by glucose tolerance tests (GTT), regardless of the diets (Fig. 4C and D). Consistently, male mice showed poor glucose tolerance than their female counterparts (2‐way ANOVA, time, *p* < 0.001, sex, *p* < 0.001, no interaction). We also examined the GTT and ITT in the female TG mice of line F26 fed with the NCD. No significant differences were noted in either test in the female TG mice compared with their WT littermate controls (Supplemental Fig. S2).

P7 TG mice of both sexes also showed no significant differences in fasting glucose or insulin compared to their WT littermates after 20 weeks on their respective diets, regardless of the diets (Table 1). High fat diet significantly increased fasting glucose levels in both female and male mice, regardless of the genotype (*p* < 0.001) (Supplemental Fig. S3 A and B). Comparing female versus male mice, male mice had higher levels of fasting blood glucose levels than their female counterparts fed with either the NCD or HFD (*p* < 0.001). High fat diet significantly increased fasting insulin levels in male mice, but not in female mice (Supplemental Fig. S3 C and D). There were no differences between TG and WT in the calculated HOMA‐IR, regardless of sex and diet (Table 1).

**Table 1:** Plasma glucose and insulin levels and HOMA‐IR of mice at termination after being fed a normal chow diet (NCD) or a high fat diet (HFD)

		Female			Male		
		WT ± SEM	TG ± SEM	*p*	WT ± SEM	TG ± SEM	*p*
Glucose 20 wk	NCD	106.9 ± 4.99	110.4 ± 4.98	ns	142.3 ± 6.54	145.3 ± 5.46	ns
(mg/dL)	HFD	139.5 ± 9.2	145.5 ± 5.38	ns	181.1 ± 9.8	170.1 ± 7.21	ns
Insulin 20 wk	NCD	0.55 ± 0.07	0.83 ± 0.11	ns	0.39 ± 0.07	0.29 ± 0.02	ns
(ng/mL)	HFD	0.48 ± 0.12	0.57 ± 0.07	ns	0.87 ± 0.17	0.79 ± 0.09	ns
HOMA‐IR 20 wk	NCD	3.67 ± 0.6	4.87 ± 0.59	ns	3.42 ± 0.59	2.73 ± 0.29	ns
	HFD	4.09 ± 0.98	5.09 ± 0.67	ns	9.82 ± 1.97	9.1 ± 1.23	ns

### Constitutive activation of TLR4 under aP2 promoter increases mRNA expression of proinflammatory genes in the white adipose tissues

**Figure 5:**
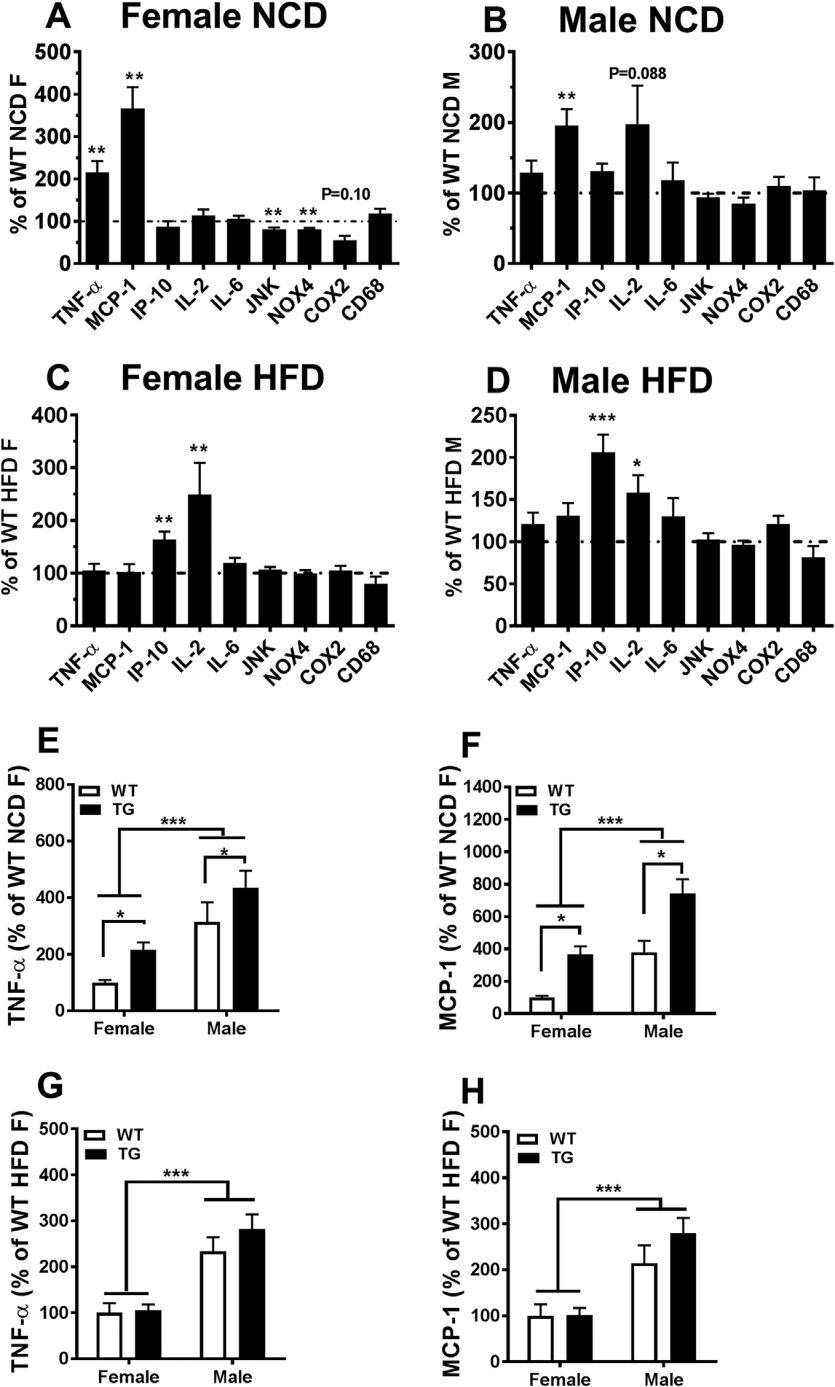
Female and male TG mice showed increased mRNA expression of select proinflammatory genes in perigonadal (PG) WAT fed either NCD or HFD. (A–D) mRNA expression of proinflammatory gene in the PG WAT of the TG mice compared with their WT littermate controls was determined in mice fed the NCD (A and B) or HFD (C and D). The data of female or male TG mice were presented as percentage relative to their same sex WT counterparts on the same diet (set to be 100%). (E–G) TNF‐α and MCP‐1 mRNA levels were compared between male and female mice fed the NCD (E and F) or the HFD (G and H). The data were presented as percentage relative to the female WT mice on the same diet (set to be 100%). Data were analyzed using the Delta Delta CT and the relative abundance of transcripts were normalized to HPRT‐1. Abbreviations are in Supplemental Table S1. Data = Mean ± SEM (*n* = 7–27). (A–D) Two tailed Student's *t*‐tests were conducted (WT vs. TG). *p < 0.05; **p < 0.01; ***p < 0.001. (E–G) 2‐way ANOVAs were conducted (sex, genotype, and interaction). No interaction between sex and genotype was found. *p < 0.05 for WT versus TG; ***p < 0.001 for female versus male.

Localized WAT inflammation has been associated with IR and the development of type 2 diabetes mellitus (T2DM) 28. The effects of ΔTLR4 expression on inflammatory and metabolic markers, as well as genotype confirmation, were assessed by semi‐quantitative RT‐PCR from the PG WAT depot. When fed with the NCD, P7 female TG mice showed a significant increase in TNFα and MCP‐1 mRNA expression compared to WT mice (115% and 266% increase, respectively; *p* < 0.05, Fig. 5A). The expression of NOX4 and JNK showed modest but significant decreases (19% and 19% decrease, respectively; *p* < 0.05, Fig. 5A). There were no significant differences in the expression of COX2 (*p* = 0.10), IP‐10, IL‐2, IL‐6, and CD68. (Fig. 5A). Similarly, P7 male TG mice fed the NCD also had increased expression of MCP‐1 (95.7% increase, *p* < 0.01). IL‐2 expression was elevated but the difference did not reach statistical significance (97.9% increase, *p* = 0.088) and no significant changes in other proinflammatory genes were noted (Fig. 5B). When comparing the differences between female and male mice, male mice had significantly higher levels of both TNF‐α (Fig. 5E and G) and MCP‐1 mRNA (Fig. 5F and H) in PG WAT than female mice, regardless of diet (*p* < 0.001). Both male and female TG mice had higher levels of TNF‐α and MCP‐1 mRNA than their WT controls only when fed the NCD (Fig. 5E and F) (*p* < 0.05). However, the percentages of induction were greater in the female TG mice than in the male TG mice, although the differences were not significant (Fig. 5A, B, E, and F).

Of note, female TG mice of line F26 also had increases in TNFα and MCP‐1 mRNA in the PG WAT compared with their WT controls when fed the NCD (Supplemental Fig. S4A).

When fed the HFD, P7 female TG mice showed significant increases in the proinflammatory mediators IP‐10 and IL‐2, compared to WT littermates (63% and 149% increase, respectively; *p* < 0.05) (Fig. 5C). Similarly, P7 male TG mice also increased IP‐10 and IL‐2 in PG WAT (106.3% and 58.4% increase, respectively; *p* < 0.05). There were no significant changes in other proinflammatory genes noted in either P7 female or male mice (Fig. 5C and D).

Plasma TNF‐α, MCP‐1, IL‐6, and other adipokine levels (total PAI‐1, adipoq, leptin, and resistin like‐α) were measured. Plasma TNFα levels were undetectable in the mice fed the NCD, but were not different between TG and WT mice fed the HFD (Table 2). No significant differences were observed for MCP‐1 levels among all samples (Table 2). Plasma total PAI‐1 levels were elevated in P7 female TG mice fed the NCD but did not reach statistical significance (*p* = 0.067). No differences were found in plasma IL‐6, adiponectin, leptin, and resistin‐like‐α between the TG and WT mice, regardless of the diets and sex.

**Table 2:** Plasma adipokine levels of mice at termination after being fed a normal chow diet (NCD) or a high fat diet (HFD)

		Female			Male		
		WT ± SEM	TG ± SEM	p*	WT ± SEM	TG ± SEM	p*
Adipq	NCD	74.79 ± 5.06	77.06 ± 3.48	ns	60.32 ± 4.06	60.51 ± 3.05	ns
(μg/mL)	HFD	16.72 ± 1.06	18.84 ± 0.89	ns	13.29 ± 0.85	13.96 ± 0.55	ns
PAI	NCD	3.28 ± 0.39	4.31 ± 0.32	0.06	3.48 ± 0.52	3.95 ± 0.39	ns
(ng/mL)	HFD	2.37 ± 0.46	2.78 ± 0.32	ns	2.26 ± 0.59	3.05 ± 0.36	ns
Leptin	NCD	4.03 ± 0.71	3.08 ± 0.45	ns	5.17 ± 1.23	6.05 ± 0.95	ns
(ng/mL)	HFD	15.83 ± 4.06	13.54 ± 2.23	ns	17.9 ± 1.88	20.69 ± 2.52	ns
Resistin	NCD	1.77 ± 0.15	1.63 ± 0.05	ns	1.38 ± 0.09	1.43 ± 0.09	ns
(ng/mL)	HFD	2.33 ± 0.35	2.38 ± 0.22	ns	2.26 ± 0.34	2.38 ± 0.17	ns
IL‐6	NCD	8.19 ± 0.8	6.83 ± 0.39	0.11	7.00 ± 0.36	6.78 ± 0.39	ns
(pg/mL)	HFD	14.19 ± 3.34	12.83 ± 1.77	ns	19.29 ± 6.67	6.55 ± 0.4	ns
MCP‐1	NCD	5.24 ± 2.15	5.61 ± 1.00**	ns	8.09 ± 3.53	7.05 ± 1.54	ns
(pg/mL)	HFD	70.16 ± 14.89	66.06 ± 8.89	ns	71.03 ± 11.31	61.31 ± 8.95	ns
TNF	NCD	UD	UD	ns	UD	UD	ns
(pg/mL)	HFD	9.56 ± 2.9	7.24 ± 1.59	ns	5.46 ± 2.45	1.32 ± 0.33	ns

*p‐Values were calculated using the Student's *t*‐test (two‐tailed) from transformed data, when needed, between WT and TG groups by diet.

**p‐Value is calculated using a binary method because over 50% of the values were below the limit of detection. Values above the detectable limit were given a value of 1 and values below the detectable limit were given a value of 0.

**Figure 6:**
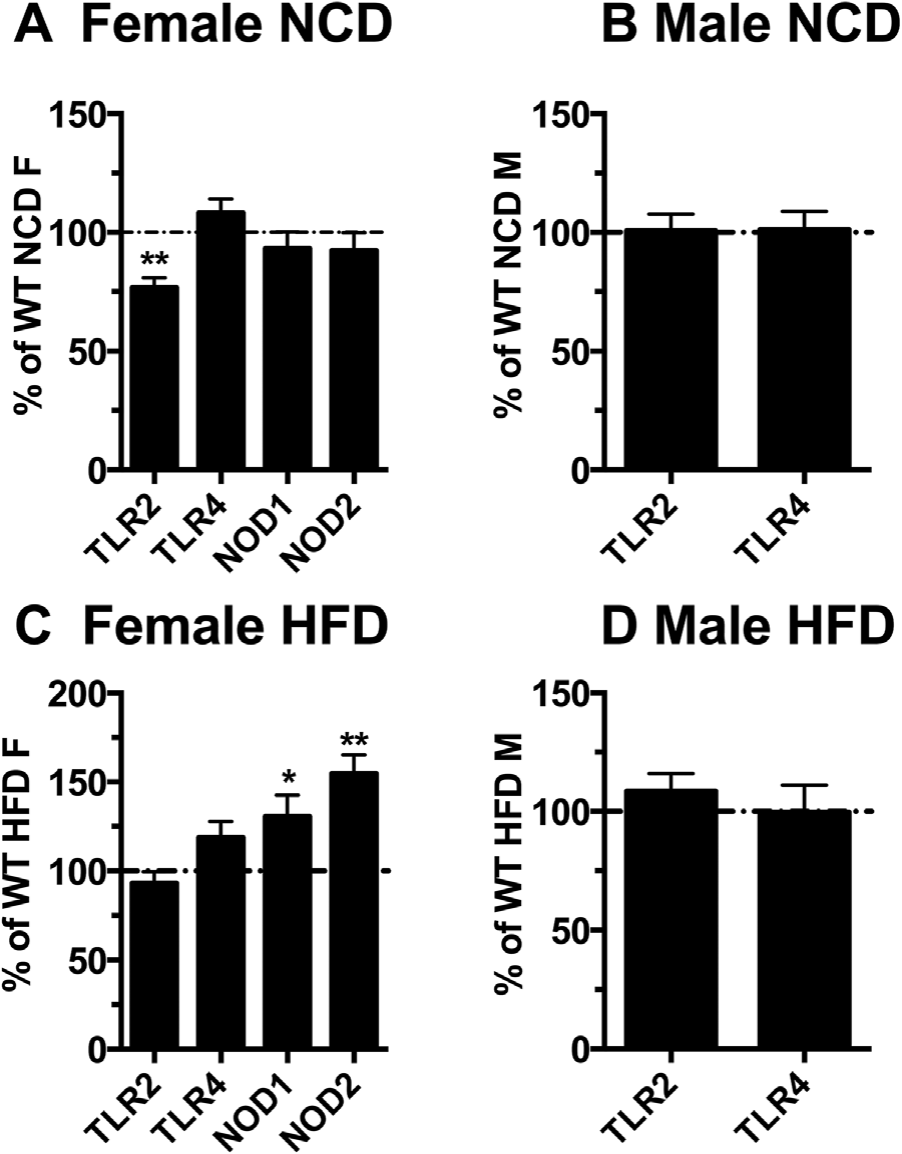
Female TG mice showed decreased TLR2 mRNA fed the NCD, but increased NOD1 and NOD2 mRNA when fed the HFD. mRNA expression of endogenous TLR4 and other pattern recognition receptors in the WAT of the TG mice compared with their littermate controls was determined in mice fed the NCD (A and B) or the HFD (C and D). Data were analyzed using the Delta Delta CT and the relative abundance of transcripts were normalized to HPRT‐1. The data of female or male TG mice were presented as percentage relative to their same sex WT counterpart (set to be 100%). Abbreviations are in Supplemental Table S1. Data = Mean ± SEM (*n* = 7–27). Two tailed Student's *t*‐tests were conducted (WT vs. TG). *p < 0.05; **p < 0.01; ***p < 0.001.

We further examined whether the expression of ΔTLR4 may affect the expression of TLR4 and other PRRs (TLR2, NOD1, NOD2). ΔTLR4 expression did not significantly affect endogenous TLR4 expression in the TG mice of either sex fed with either diet (Fig. 6). ΔTLR4 expression significantly decreased TLR2 expression in the P7 females fed the NCD (23%, *p* < 0.05) (Fig. 6A). ΔTLR4 expression significantly increased expression of NOD1 and NOD2 (31%, and 54% increase, respectively; *p* < 0.05) with no significant effect on TLR2 expression (Fig. 6C) in the P7 females fed the HFD. There was no change in TLR2 mRNA expression in the males fed either diet (Fig. 6B and D). There were no changes in other PRR expression between the female TG and WT mice of line F26 fed the NCD (Supplemental Fig. S4B).

### Constitutive activation of TLR4 under aP2 promoter increases expression of negative regulators of TLR4 signaling in females of transgenic mice fed a HFD

**Figure 7:**
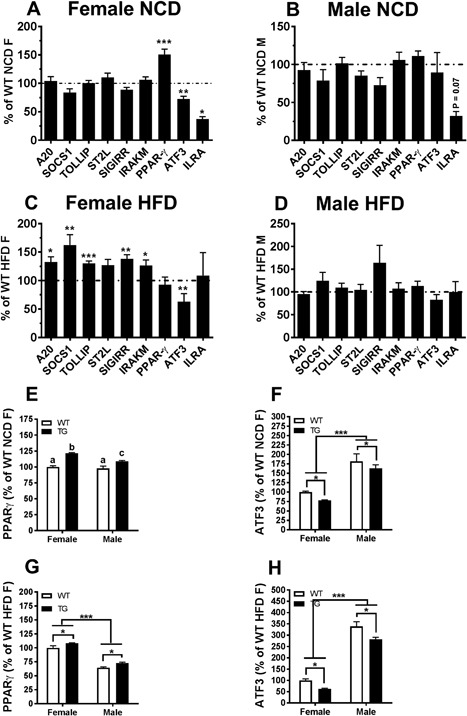
Female TG mice fed the HFD showed increased mRNA expression of PPARγ and other negative regulators of TLR4. (A–D) mRNA expression of select negative regulators of TLR4 signaling in the PG WAT of TG mice compared with their WT littermate controls was determined in mice fed the NCD (A and B) or HFD (C and D). The data of female or male TG mice were presented as percentage relative to their same sex WT counterpart on the same diet (set to be 100%). (E–G) PPARγ and ATF3 mRNA levels were compared between male and female mice fed the NCD (E and F) or the HFD (G and H). The data were presented as percentage relative to the female WT mice on the same diet (set to be 100%). Data were analyzed using the Delta Delta CT and the relative abundance of transcripts were normalized to HPRT‐1. Abbreviations are in Supplemental Table S1. Data = Mean ± SEM (*n* = 7–27). (A–D) Two tailed Student's *t*‐tests were conducted (WT vs. TG). *p < 0.05; **p < 0.01; ***p < 0.001. (E–G) Two‐way ANOVAs were conducted (sex, genotype, and interaction). (E) There was a significant sex by genotype interaction for the expression of PPAR‐γ in mice fed a NCD (*p* < 0.05). Groups with differing letters indicate a significant difference (*p* < 0.05). (F–H) No interaction between sex and genotype for other variables was found. *p < 0.05 for WT versus TG. ***p < 0.001 for female versus male.

Constitutive activation of TLR4 is known to desensitize the response to the TLR4 agonist LPS in an organism 29. The constitutively active nature of ΔTLR4 in the TG mice may produce such desensitization to TLR4 activation by increasing expression of negative regulators of TLR4 signaling. Such negative regulators of the TLR4 pathway include ATF3, PPARγ, A20, SOCS1, TOLLIP, SIGGIR, IRAKM, IL‐RA, and IL‐10 29, 30, 31, 32, 33. We examined mRNA expression of these negative regulators in the PG WAT depot of mice fed the NCD or HFD. P7 female TG mice fed the NCD exhibited a significant increase of PPARγ mRNA (50%, *p* < 0.05, Fig. 7A) compared to their WT littermates. However, mRNA expression of IL‐RA and ATF3 was suppressed by 63% (*p* < 0.05) and 27.5%, respectively (*p* < 0.01). No significant differences were seen in the other negative regulators of the TLR4 pathway measured (A20, SOCS1, TOLLIP, SIGGIR, IRAKM). In contrast, P7 female TG mice fed the HFD had significant increases in the mRNA expression of multiple negative regulators of the TLR4 pathway, including A20, SOCS1, TOLLIP, SIGIRR, and IRAKM, compared to those in their WT littermates (32%, 62%, 30%, 38%, and 26% increase, respectively; *p* < 0.05) (Fig. 7C). The expression of the negative regulator ST2L was also increased (32%), but it did not reach statistical significance (*p* = 0.053). In contrast, ATF3 mRNA was significantly suppressed (37%, *p* < 0.05). None of these negative regulators of TLR4 signaling were changed in the male TG mice compared to their WT controls, regardless of the diet (Fig. 7B and D). When comparing the differences between female and male mice, under the NCD female TG mice had higher PPARγ mRNA than female WT mice. Male TG mice also had higher PPARγ mRNA than male WT mice (Fig. 7E). However, PPARγ up‐regulation in female TG mice was higher than male TG mice (*p* < 0.01). Under the HFD, female mice had higher PPARγ mRNA than their male counterparts (Fig. 7G). TG mice of both sexes had higher PPARγ mRNA than their same sex WT controls (Fig. 7G). Regardless of diet, female mice had lower ATF3 mRNA than their male counterparts (*p* < 0.001 for both diets). TG mice had lower ATF3 mRNA than their WT controls, regardless of sex (*p* < 0.05 for both diets). None of the negative regulators of TLR4 signaling had altered expression levels in the female TG mice of line F26 when fed the NCD (Supplemental Fig. S4C).

## Discussion

Previous studies have shown SFAs can activate TLR4, inducing sterile inflammation and IR 34. The role of sterile inflammation induced by a HFD (typically 60% kcal from fat) in the development of IR has been investigated by using murine KO models of proinflammatory genes (TLRs, NF‐κB subunits, cytokines, etc.). For instance, TLR4 or TLR2 KO mice were partially protected from the HFD‐induced inflammation and IR 18, 19, 35, 36. These mice tend to be more insulin sensitive as measured by ITT or have improved insulin signaling. The protection of TLR4 mutant or KO mouse models from HFD‐induced IR has been attributed to decreased inflammation particularly in WAT 18, 19, 36 and suggests that HFD‐induced IR is at least in part mediated through TLR4 signaling pathways; this raised another question: whether in vivo activation of TLR4 would be sufficient to induce IR? However, direct evidence that TLR4 activation in WAT promotes development of IR has not been demonstrated. Therefore, the purpose of this study was to investigate whether the activation of TLR4 in the adipose tissues in a ligand independent and adipose‐specific manner induces metabolic changes, such as obesity and insulin resistance. Accordingly, we have generated the transgenic mice expressing the constitutively active ΔTLR4 in adipose tissue under the control of the aP2 promoter (aP2‐ΔTLR4). Phenotypes of these transgenic mice may reveal whether the specific ligand independent activation of TLR4 in adipose tissue is sufficient to induce IR. One limitation of using aP2 promoter to drive the transgene expression in adipose tissue is that the transgene can be expressed not only in adipose tissue but also in macrophages that reside many tissues and organs. Thus, the interpretation of the results presented here is made in recognition of this limitation.

Based on the two transgenic lines that were generated and characterized, we found that aP2‐driven expression of constitutively active TLR4 was not sufficient to induce systemic insulin resistance, despite being sufficient to induce elevated inflammation in WAT. Moreover, female, but not male, mice of one line of transgenic mice exhibited greater insulin sensitivity and decreased body weight when fed a NCD. When fed with a HFD, female mice from this line exhibited no difference in body weight and insulin sensitivity compared to WT littermate controls, possibly due to robust upregulation of negative regulators of TLR4 signaling, PPARγ, A20, SOCS1, TOLLIP, SIGGIR, IRAKM, and IL‐10 29, 30, 31, 32, 33, which may have dampened inflammation induced by the constitutive expression of ΔTLR4 under HFD.

It is well known that adiposity and body weight gain can greatly influence insulin sensitivity 37, 38. Therefore, the improved insulin sensitivities may be associated with the reduced body weight and adiposity found in the P7 female TG mice fed the NCD. Reduced body weight and adiposity may be due to increased energy expenditure and/or changes in food intakes caused by ΔTLR4‐mediated inflammation as enhanced inflammation is known to cause a substantial increase in energy expenditure 39. This increase in energy expenditure and metabolic rate is due to the induction of fever and the activation of immune cells. It is noteworthy that transgenic mice with over‐expression of p65 (a subunit of NFκB) 40 or IKKβ 41 using the aP2 promoter exhibited improved insulin sensitivity despite increased inflammation. Tang et al. 40 reported that mice with aP2 promoter driven p65 expression or mice with a global KO of p50 were protected from the diet‐induced IR and impaired glucose tolerance despite increased systemic inflammation and increased localized WAT inflammation. Both adipose‐specific p65 and IKKβ transgenic mice showed increased metabolic rate, which might have contributed to the improved insulin sensitivity 40, 41. However, we did not detect significant differences in energy expenditure in the TG mice compared to the WT controls (Supplemental Fig. S5). On the other hand, inflammation affects food intake. The cytokines, TNF‐α and IL‐1α, have been shown to suppress food intake 42. The decrease in body weight in P7 female TG mice fed the NCD is associated with a reduction in the food intake, which could have been caused by cytokines induced by the transgene‐induced inflammation. There was increased TNF‐α mRNA in WAT in the P7 female TG mice; however, whether this increase led to elevated circulating levels of TNF‐α has not been confirmed because plasma TNF‐α levels were not detectable in mice fed the NCD. Together, our results and other studies with IKKβ or p65 transgenic mice 40, 41 suggest an intriguing possibility that inflammation in white adipose tissue may have indirect effects in improving insulin sensitivity through reducing food intake and/or body weight, which can counteract impairment of insulin signaling caused by proinflammatory signals.

It should be noted that although the activation of TLR4, IKKβ, or p65 all leads to increased expression of proinflammatory gene products, IKKβ or p65 are both downstream components of TLR4. TLR4 can activate multiple downstream signaling pathways (i.e., PI3K, NOX, micro RNA anti‐inflammatory pathways), which may not be affected by the overexpression of further downstream signaling components, such as IKKβ or p65. Therefore, divergent phenotypes of the ΔTLR4 transgenic mice with IKKβ or p65 transgenic mice 40, 41 are expected. All three transgenic mice showed increased inflammation in adipose tissues but with no impairment in insulin sensitivity, suggesting that the contribution of decreased body weight to improved insulin sensitivity found in those transgenic animals might have overridden the impairment of insulin signaling caused by increased inflammation in WAT.

The compensatory mechanism of the immune system to TLR4 activation by LPS has been well documented. In vivo, a second exposure to LPS (TLR4 ligand) exhibits a decreased inflammatory response compared to the first exposure, a phenomenon known as “endotoxin tolerance.” Not only proinflammatory mediators but also negative regulators of the TLR pathway (e.g., IRAKM and SIGIRR) are in part responsible for endotoxin tolerance. Our transgenic mice with the constitutive activation of TLR4 in adipose tissue might have altered local and systemic inflammatory status due to up regulation of the negative regulators of the TLR4 pathway as a result of transgene ΔTLR4 expression. This hypothesis seems more apt to describe the up‐regulation of the negative regulators in the female TG mice fed the HFD than female mice fed the NCD. Whether HFD alone in non‐transgenic mice can up‐regulate the expression of the negative regulators will need to be determined in the future. While female TG mice fed the NCD had increased expression of PPARγ, it is not known whether this or other negative regulators of TLR4 are also up‐regulated in IKKβ and p65 transgenic mice. It is possible that the signaling pathways leading to the expression of negative regulators might have diverged from the upstream of IKKβ.

As only female TG mice fed the NCD showed significant differences in body weight, food intake, and insulin sensitivity compared to their WT controls, we compared sex differences in metabolic markers and mRNA expression of proinflammatory genes and negative regulators of TLR4 signaling. Regardless of diet and genotype, male mice had poor insulin sensitivity and glucose tolerance, higher proinflammatory TNF‐α and MCP‐1 mRNA, and higher ATF3 mRNA compared to their female counterparts. HFD‐fed female had higher PPARγ than their male counterparts, regardless of genotype and TG mice had higher PPARγ mRNA than the WT controls. NCD‐fed female TG mice had higher PPARγ than male TG mice fed the NCD, but there were no differences in PPARγ mRNA between female and male WT mice fed the NCD. HFD‐fed male mice showed both insulin resistant and glucose intolerant than male mice fed the NCD whereas HFD‐fed female mice were only more glucose intolerant compared to the female mice fed the NCD. Our findings of the sex differences in metabolic markers in mice are consistent with previous reports 43, 44. It seems that higher levels of proinflammatory gene expression are associated with poor metabolic biomarkers observed in a sex‐specific manner. Future studies are needed to explore the roles of negative regulator PPARγ and ATF3 in TLR4‐mediated inflammation and associated metabolic impact.

It is noted that only one of the two transgenic lines (line P7) in our study showed reduced body weight and food intakes with improved insulin sensitivities. The phenotypes of the transgenic mice are known to be affected by the location of the insertion of the transgene. It is possible that differences in the phenotypes of our two lines are affected by their insertion sites in the genome. However, both lines of the transgenic mice did not show impaired insulin resistance.

In summary, our results demonstrate that ectopic expression of constitutively active TLR4 driven by aP2 promoter induces inflammation in white adipose tissue; however, it does not induce systemic insulin resistance. Interestingly, female transgenic mice fed the NCD showed improved insulin sensitivity compared to their WT littermate controls. It should be confirmed in the future whether high‐fat diet induced insulin resistance may require other signaling pathways and whether TLR4‐mediated inflammation in white adipose tissue may have beneficial effects in improving insulin sensitivity, possibly through reducing food intake and/or body weight, which can counteract impairment of insulin signaling caused by proinflammatory signals. Our results that adipose‐specific TLR4‐mediated sterile inflammation does not impair insulin sensitivity and may actually improve it, underscores that our understanding of roles of TLR4‐mediated inflammation on insulin sensitivity is incomplete.

## Materials and Methods

### Construction of constitutively active TLR4 under aP2 promoter expression plasmid

The Toll in *Drosophila* embryo lacking the extracellular leucine‐rich repeat domain produces a dominant gain‐of‐function mutant of the TOLL protein 45. The dominant gain‐of‐function mutants do not require the ligand for activation. Likewise, we previously found that the deletion of the leucine‐rich repeats in the N‐Terminus of the murine TLR4 produced a similar gain‐of‐function or a constitutively active form of TLR4, named ΔTLR4 46, as shown in Figure 1A. The aP2 promoter‐driven ΔTLR4 construct was generated by ligating the 5.4 kb aP2 promoter/enhancer, kindly provided by Dr. Bruce Spiegelman (Dana‐Farber Cancer Institute, Boston, MA), with HA‐ΔTLR4 fragment and a sequence of bovine growth hormone polyadenylation signal sequence. HA‐ΔTLR4 fragment was prepared by polymerase chain reaction (PCR) using pcDNA3.1‐HA‐ΔTLR4 46 as a template with a 5′ primer sequence including a SmalI site and a Kozak consensus sequence, paired with a 3′ primer sequence including a ScaII site. The schematic design is depicted in Figure 1B. TG mice were generated using pronuclear injection techniques into B6D2F1 fertilized eggs (Mouse Biology Program, University of California, Davis, CA). Two lines of mice were established, referred to as P7 and F26. Mice were genotyped by analysis of tail genomic DNA. All animal procedures were approved by the University California, Davis's Institutional Animal Care and Use Committee.

### Mice

Mice were maintained under 12‐h light and dark cycles in a specific pathogen free facility, and were fed either a NCD (PicoLab 5053, Lab Diet, Brentwood, MO) or a HFD, 60% kcal from fat (product # D12492, Research Diets, NJ). A NCD was used as the control in many KO mice studies 18, 19, 40, 41, 47, 48, 49, 50 and was used instead of a semi‐purified low fat diet to be consistent with these studies. Body weight was measured weekly starting at ∼5 weeks of age until the mice were 25 weeks of age. Food intake was measured between 21 and 25 weeks of age. Fasting glucose levels were measured by OneTouch Ultra 2 glucometer (LifeScan, Milpitas, CA) after the animals were fasted overnight (12–14 h). Fasting insulin levels were measured using the Ultra Sensitive mouse insulin ELISA (Crystal Chem, Inc., Downers Grove, IL). HOMA‐IR was calculated as HOMA‐IR = Fasting insulin (mIU/L) × Fasting glucose (mg/dL)/405 51.

At termination, fasted mice were anesthetized with isoflurane before terminal blood was collected by retro‐orbital bleeds or cardiac punctures. The mice were euthanized by cervical dislocation; perigonadal (PG), retroperitoneal (RP), and subcutaneous (SQ) WAT, and brown adipose tissue (BAT) were collected, in addition to the liver and gastrocnemius muscle. Tissues were immediately snap frozen in liquid nitrogen.

### Glucose tolerance tests and insulin tolerance tests

Glucose tolerance tests (GTTs) and insulin tolerance tests (ITTs) were performed as previously described 49. Briefly, GTTs were performed at 16 weeks of age (1 g/kg of body weight of dextrose) after an overnight fast on mice fed a NCD or a HFD. Dextrose (Hospira, Inc., Lake Forest, IL) was diluted in injection grade water and administered by oral gavage (Solomon Scientific, San Antonio, TX). ITTs were performed at 17 weeks of age, after a 5 h fast. Humulin R insulin (Eli Lilly and Co., Indianapolis, IN) was diluted in 0.9% saline (Phoenix Pharmaceutical, Inc., Burlingame, CA) and administered by intraperitoneal injection at 0.75 U/kg of body weight and glucose values were measured as using OneTouch Ultra 2 glucometer.

### Measurement of adipokines

Plasma levels of leptin, monocyte chemotactic protein‐1 (MCP‐1), tumor necrosis factor‐α (TNF‐α), total plasminogen activator inhibitor‐1 (total PAI‐1), resistin‐like α, interleukin‐6 (IL‐6), and adiponectin were measured using Milliplex bead‐based immunoassays (Millipore, Billerica, MA).

### Quantitative real‐time PCR

Total RNA was isolated from various tissues using the RiboPure RNA isolation kit (Life Technologies, Inc., Grand Island, NY) per manufacturer's instructions. RNA integrity was analyzed using an Agilent 2100 Bioanalyzer (Agilent Technologies, Santa Clara, CA). RNA concentration was determined using a NanoDrop Spectrophotometer (Thermo Fisher Scientific, Waltham, MA) and cDNA was synthesized from 1 μg of total RNA using Superscript III cDNA synthesis kit (Life Technologies, Inc.). Semi‐quantitative PCR was performed using an ABI prism 7900 PCR instrument with gene‐specific Taqman gene expression assays (Supplemental Table S1). The relative abundance of transcripts were normalized to hypoxanthine‐guanine phosphoribosyltransferase‐1 (HPRT‐1). Data were analyzed using the Delta Delta CT method as previously described 52.

### Immunoprecipitation analysis

Tissues were homogenized in M‐PER (Thermo Fisher Scientific) with the protease inhibitor HALT (Thermo Fisher Scientific). Protein lysates were centrifuged at 10,000*g* at 4°C for 10 min and 16,000*g* at 4°C for 10 min to clear lipids. Protein concentrations of the samples were determined with Bradford Plus Protein Assay reagent (Thermo Fisher Scientific). Immunoprecipitations were performed with 0.5–5 mg of protein lysates using anti‐HA affinity matrix (Roche, Indianapolis, IN) overnight. The anti‐HA affinity matrix was washed three times (once with M‐PER and twice with PBS) before 2× sample loading buffer was added. The samples were heated for 3 min at 100°C then subjected to electrophoresis on 10% Tris‐Glycine gels, and transferred to polyvinylidene difluoride (PVDF) membranes (Bio‐Rad Laboratories, Hercules, CA). The membrane was blocked in 5% non‐fat dry milk in tris‐buffered saline with 0.1% tween 20 (TBS‐T) for 1 h. The membrane was incubated with anti‐HA (Cell Signaling, Danvers, MA) in 2.5% non‐fat dry milk overnight. HRP conjugated secondary antibodies (Amersham, Pittsburgh, PA) were incubated for 1 h in 2.5% of non‐fat dry milk. The band was visualized using the chemiluminescent substrate ELC Plus (Amersham, Pittsburgh, PA).

### Bone marrow‐derived macrophages

Bone marrow‐derived macrophages (BMDM) were isolated as described 53. Mice were euthanized by CO_2_ asphyxiation. Intact femurs were collected in sterile PBS. Femurs were sterilized in 70% ethanol for 2 min before the bone cavities were flushed with cold PBS. The cells were filtered through a 50 μM cell strainer and collected by centrifugation. The cells were resuspended in DMEM F‐12, 10% FBS, 15% L‐929 conditioned medium (produced as described 53), 1% penicillin/streptomycin (Life Technologies, Inc.), and 10 μg/mL plasmocin (Invivogen, San Diego, CA). BMDM were considered differentiated on day eight at which time total RNA was collected.

### Statistical analysis

Results are presented as mean ± SEM. For temporal outcomes, such as body weight gain, GTTs and ITTs, ANOVA with repeated measures (Proc Mixed) was conducted to compare differences between genotypes. Other outcomes such as gene expression and tissue masses were analyzed using the Student's *t*‐test (two‐tailed) (SAS, Windows Release 9.2 [Cary, NC]). Two‐way ANOVA was conducted to identify the interaction among sex, genotype, and diet using GraphPad Prism 7 (GraphPad Software, Inc.). Appropriate transformations were made when basic testing assumptions were violated (e.g., log variable). Outlier testing was conducted using the ROUT method (GraphPad Prism Version 6, La Jolla, CA). Values of plasma cytokines that were undetectable were replaced using the 2/3 of the assay's limit of detection. Statistical significance was determined as *p* < 0.05 and statistical trends was defined as *p* < 0.10.

## Conflict of Interest

All authors have no conflict of interest to disclosure.

## Supporting information

Additional supporting information may be found in the online version of this article at the publisher's web‐site.

**Figure S1**. Body weight gain over time and terminal fat pad weights in a second line (F26) TG mice compared with their littermate controls.
**Figure S2**. Insulin and glucose tolerance tests in the female TG mice of F26 compared to their WT littermate controls when fed a normal chow diet.
**Figure S3**. Diet‐induced differences in fasting blood glucose and insulin levels. Fasting blood glucose (A and B) and insulin (C and D) were compared between female (A and C) and male (B and D) mice based on the genotype.
**Figure S4**. mRNA expression of TLR4 gene products (A), other pattern recognition receptors (PRR) (B), and negative regulators of TLR4 (C) in the female TG mice of F26 line compared with their WT littermate controls when fed a normal chow diet.
**Figure S5**. Respiratory exchange ratio (RER) (A) and energy expenditure (EE) (B) of female mice of F26 line fed a NCD did not differ between genotypes at 24 week of age.
**Table S1**. Taqman Gene Expression Assays‐on‐Demand (Part#43331182).
