# Supplementary material for: Transgenic mice with ectopic expression of constitutively active TLR4 in adipose tissues do not show impaired insulin sensitivity
Source: Immun Inflamm Dis. 2017 Aug 4;5(4):526–40. doi: 10.1002/iid3.162 (PMC5691308; doi:10.1002/iid3.162)
Supplement: Supplementary file 1 — Figure S1. Body weight gain over time and terminal fat pad weights in a second line (F26) TG mice compared with their littermate controls. Figure S2. Insulin and glucose tolerance tests in the female TG mice of F26 compared to their WT littermate controls when fed a normal chow diet. Figure S3. Diet‐induced differences in fasting blood glucose and insulin levels. Fasting blood glucose (A and B) and insulin (C and D) were compared between female (A and C) and male (B and D) mice based on the genotype. Figure S4. mRNA expression of TLR4 gene products (A), other pattern recognition receptors (PRR) (B), and negative regulators of TLR4 (C) in the female TG mice of F26 line compared with their WT littermate controls when fed a normal chow diet. Figure S5. Respiratory exchange ratio (RER) (A) and energy expenditure (EE) (B) of female mice of F26 line fed a NCD did not differ between genotypes at 24 week of age. Table S1. Taqman Gene Expression Assays‐on‐Demand (Part#43331182). [file IID3-5-526-s001.doc]

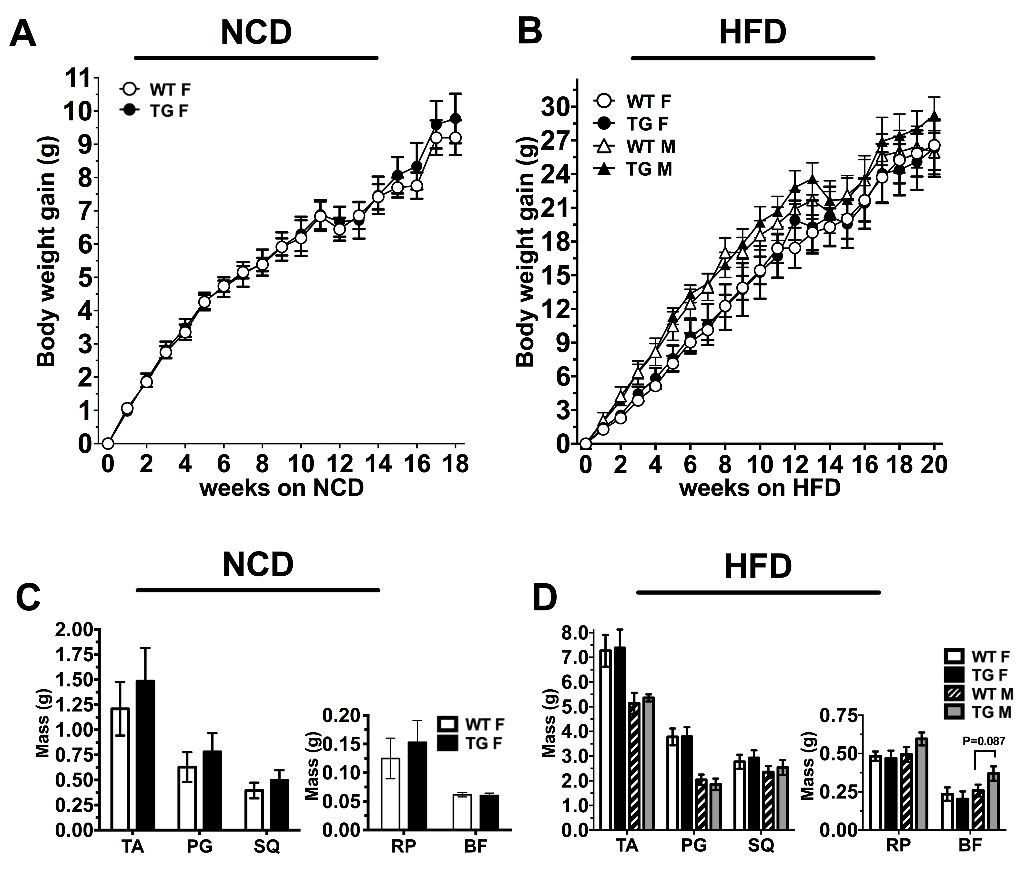


**Supplemental Figure 1** Body weight gain over time and terminal fat pad weights in a second line (F26) TG mice compared with their littermate controls. (A, B) female (F) TG and their WT littermate controls were put on either a NCD or HFD starting from 4-6 weeks of age. Body weights were recorded. The data was analyzed by repeated measures ANOVA of genotype*time. (C, D) tissue weights of various fat pads were measured at termination. RP and BF data are on separate graphs to better display the differences. BF, brown fat; PG, perigonadal fat; RP, retroperitoneal fat; SQ, subcutaneous fat; TA, sum of total adipose. Error bars are ±SEM (n = 14 – 16 per group for weight gain) (n = 7 – 9 per group for tissues weights) A few error bars are hidden by the symbols. Two tailed Student’s t-tests were conducted. (WT vs. TG). *, p < 0.05; **, p < 0.01; ***, p < 0.001

**Supplemental** Figure 2 Insulin and glucose tolerance tests in the female TG mice of F26 compared to their WT littermate controls when fed a normal chow diet. (A) Insulin tolerance tests. (B) Glucose tolerance tests. Error bars are ±SEM (n = 14 - 16). ANOVA with repeated measures were used. No significant differences were detected.

**Supplemental** Figure 3 mRNA expression of TLR4 gene products (A), other pattern recognition receptors (PRR) (B), and negative regulators of TLR4 (C) in the female TG mice of F26 line compared with their WT littermate controls when fed a normal chow diet. Data were analyzed using the Delta Delta CT and the relative abundance of transcripts were normalized to HPRT-1. Abbreviations are in Supplemental Table 1. Error bars are ± SEM (n = 7 – 9 per group). Two-tailed student t-tests were conducted. (WT vs. TG). *, p < 0.05; **, p < 0.01; ***, p < 0.001

**Supplemental Figure 4** Respiratory exchange ratio (RER) (A) and energy expenditure (EE) (B) of female mice of F26 line fed a NCD did not differ between genotypes at 24 week of age. Mean ± SEM (n = 6 per group). Two tailed Student’s t-tests were conducted. (WT vs. TG). No significant differences were detected.

**Supplemental Table** 1**.** Taqman Gene Expression Assays-on-Demand (Part#43331182)*

| Gene ID | Gene Name | Assay ID | Amplicon Size |
| --- | --- | --- | --- |
| Tnfaip3 | tumor necrosis factor alpha-induced protein 3 (A20) | Mm00437121_m1 | 76 |
| Adipoq | adiponectin(Adipoq) | Mm00456425_m1 | 74 |
| Atf3 | activating transcription factor 3(ATF3) | Mm00476032_m1 | 61 |
| Cd68;- | CD68 (CD68) | Mm00839636_g1 | 86 |
| Ptgs2 | prostaglandin-endoperoxide synthase 2(COX2) | Mm00478374_m1 | 80 |
| Hprt | hypoxanthine guanine phosphoribosyl transferase(HPRT1) | Mm00446968_m1 | 65 |
| Il1b | interleukin 1 beta(IL-1B) | Mm00434228_m1 | 90 |
| Il2 | interleukin 2(IL-2) | Mm00434256_m1 | 82 |
| Il6 | interleukin 6(IL-6) | Mm00446190_m1 | 78 |
| Il1rn | interleukin 1 receptor antagonist(IL-RA) | Mm01337566_m1 | 66 |
| Cxcl10 | chemokine (C-X-C motif) ligand 10(IP-10) | Mm00445235_m1 | 59 |
| Irak3 | interleukin-1 receptor-associated kinase 3(IRAKM) | Mm01164306_m1 | 74 |
| Mapk8 | mitogen-activated protein kinase 8(JNK) | Mm00489514_m1 | 99 |
| Lep | leptin(Lep) | Mm00434759_m1 | 73 |
| Ccl2 | chemokine (C-C motif) ligand 2(MCP-1) | Mm00441242_m1 | 74 |
| Nod1 | nucleotide-binding oligomerization domain containing 1(NOD1) | Mm00805062_m1 | 63 |
| Nod2 | nucleotide-binding oligomerization domain containing 2(NOD2) | Mm00467543_m1 | 78 |
| Nos2 | nitric oxide synthase 2(NOS2) | Mm00440485_m1 | 70 |
| Nox4 | NADPH oxidase 4(NOX4) | Mm00479246_m1 | 72 |
| Pparg | peroxisome proliferator activated receptor gamma(PPAR-Y) | Mm00440945_m1 | 105 |
| Retnla | resistin like alpha(Retnla) | Mm00445109_m1 | 86 |
| Sigirr | single immunoglobulin and toll-interleukin 1 receptor (TIR) domain(SIGIRR) | Mm00491700_m1 | 84 |
| Socs1 | suppressor of cytokine signaling 1(SOCS1) | Mm01342740_g1 | 133 |
| Il1rl1 | interleukin 1 receptor-like 1(ST2L) | Mm01233979_m1 | 75 |
| Tlr2 | toll-like receptor 2(TLR2) | Mm00442346_m1 | 69 |
| Tlr4 | toll-like receptor 4(TLR4) | Mm00445273_m1 | 87 |
| Tnf | tumor necrosis factor(Tnf) | Mm00443258_m1 | 81 |
| Tollip | toll interacting protein(TOLLIP) | Mm00445841_m1 | 64 |
| Ucp2 | uncoupling protein 2(UCP2) | Mm00627599_m1 | 137 |

*Purchased from Life Technologies, Inc., Grand Island, NY
